# Berberine improves negative symptoms and cognitive function in patients with chronic schizophrenia via anti-inflammatory effect: a randomized clinical trial

**DOI:** 10.1186/s13020-023-00746-4

**Published:** 2023-04-17

**Authors:** Zhengping Pu, Hui Wen, Hongxia Jiang, Qingmei Hou, Hui Yan

**Affiliations:** 1 Department of Psychiatry, Kangci Hospital of Jiaxing, No. 3118 Huancheng North Road, Tongxiang, 314500 Zhejiang China; 2 Shanghai Mental Health Center, Shanghai Jiao Tong University School of Medicine, Minhang, Shanghai, 201108 China; 3 Department of Traditional Chinese Medicine, Second People’s Hospital of Tongxiang, Tongxiang, 314500 Zhejiang China; 4 Department of Clinical Psychology, The Second Specialized Hospital of Hegang, Hegang, 154102 Heilongjiang China; 5 Department of Psychiatry, Second People’s Hospital of Taizhou, Taizhou, 317200 Zhejiang China

**Keywords:** Berberine, Negative symptom, Cognitive impairment, Chronic schizophrenia, Inflammation

## Abstract

**Background:**

Based on the neuroinflammation hypothesis in schizophrenia and known anti-inflammatory effects of berberine, the aim of the present study is to investigate the efficacy of berberine in treating negative symptoms and cognitive deficits in adult patients with chronic schizophrenia.

**Methods:**

Enrolled participants were randomized to receive berberine or placebo for 3 months. The Scale for the Assessment of Negative Symptoms (SANS), Trail-making Test A (TMT-A), Trail-making Test B (TMT-B), and Hopkins Verbal Learning Test (HVLT) were used to evaluate the negative symptoms and cognitive function at four-time points (baseline, 1st, 2nd, and 3rd month). Serum levels of interleukin-1β (IL-1β), interleukin-6 (IL-6), and tumor necrosis factor-α (TNF-α) were used as inflammatory markers. 106 patients with per-protocol were analyzed, 56 in the experimental (berberine) group and 50 in the control (placebo) group.

**Results:**

From baseline to month 3, patients receiving berberine demonstrated a decrease in total scores on clinical scales SANS, TMT-A and TMT-B and showed a serum level reduction of IL-1β, IL-6 and TNF-α comparing with patients in the control group (P < 0.05). There were positive correlations between the change of serum IL-1β level and the change of SANS (r = 0.210, P = 0.039), TMT-A (*r* = 0.522, *P* < 0.001), and TMT-B (*r* = 0.811, P < 0.001); between the change of serum IL-6 level and the change of TMT-A (*r* = 0.562, *P* < 0.001), and TMT-B (*r* = 0.664,* P* < 0.001); between the change of serum TNF-α level and the change of TMT-B (*r* = 0.472, *P* < 0.001) after berberine treatment.

**Conclusions:**

Berberine is an anti-inflammatory agent that can potentially mitigate the negative symptoms and cognitive deficits in patients with schizophrenia.

## Background

The negative symptoms and cognitive impairments are considered the core clinical features of schizophrenia which can be especially pronounced in patients at chronic stages of the illness. The negative symptoms can be manifested as avolition, anhedonia, affective blunting, alogia, and social withdrawal. The primary cognitive impairments refer to domains of attention and processing speed, executive function, and memory [1]. The negative symptoms and cognitive impairments are the leading causes of the high disability rate and substantial financial burdens of chronic schizophrenia as they synergistically impair patients’ social function and daily life [2, 3].

The negative symptoms and cognitive impairments are strongly correlated [4]. It has been hypothesized that these clinical features have overlaps at the pathophysiological level, clinical development, and prognosis [5]. Some scholars found about a 20% overlap of clinical features between these two syndromes [6]. For example, both involve impairments of attention, thinking logic, and the structure of the prefrontal cortex [1]. The hypofunction of glutamatergic neurons in the prefrontal cortex [7], decreased concentration of dopamine in the mesocortical system [1], and excessive expression of the histamine-3 receptor [8, 9] are considered as the common biochemical mechanisms shared by these two clinical domains.

Besides the above mechanisms, the pathophysiology of schizophrenia is strongly associated with neuroinflammation [12]. The dysimmunity and low chronic inflammation have gradually become the new potential targets of chronic schizophrenia [13]. It is reported that inflammatory markers from cellular to molecular levels have changed in chronic schizophrenia, which manifested via the increased active microglia, impaired astrocytes, and exaggerated expression of kinds of cytokines. Studies have demonstrated the critical role of inflammatory factors, including C-creative protein, IL-1β, IL-6, TNF-α, and interferon in neuroimmunology, neuroplasticity, neuroendocrine and signal transduction [14]. The research findings by far have shown the close relationships between these inflammatory factors and the psychosis symptoms, especially the catatonic behaviors [15], negative symptoms [16] and cognitive impairment [17].

Therefore, treating negative symptoms and addressing illness-associated cognitive impairments are important clinical goals. Over the past several decades of clinical research, there has been significant progress in providing a wide spectrum of medications to treat schizophrenia. However, recent meta-analyses converge on the notion that the acute efficacy of antipsychotics is modest [10], especially in attempts to treat cognitive impairments and negative symptoms. Theoretically, the atypical antipsychotics can improve these two clinical domains for their relatively low influence on dopamine receptors in the mesocortical system and the inhibiting effects on 5-hydroxytryptamine-2 receptors and histamine receptors [1]. However, the actual therapeutic effects of the atypical antipsychotics on negative symptoms and cognitive impairments are minimal [11]. In addition, the atypical antipsychotics also possess some adverse reactions such as metabolic syndrome, excessive sedation, postural hypotension, arrhythmia, dry mouth, constipation etc. [1].

In line with the neuroinflammation hypothesis, previous studies suggested that some antibiotics and non-steroidal anti-inflammatory drugs including minocycline, aspirin, and celecoxib can ameliorate the negative symptoms and cognitive impairments of schizophrenia [18]. However, the clinical utility of these medications is limited due to the known adverse reactions such as dysbacteriosis, gastric mucosal lesion, and stomach bleeding. Therefore, there is an urgent need for new pharmacological agents with better side-effect profiles and improved efficacy.

Berberine is an alkaloid chemical extracted from the traditional Chinese medicine *coptis chinensis* (Huanglian) or *cortex phellodendri* (Huangbai). They are widely used in China as an antidiarrheal drug with its non-specific anti-inflammatory effects, affordability, and safety. There are several relevant clinical trials that used berberine in schizophrenic patients, however, these studies focused on berberine application in preventing the metabolic disturbances resulted from antipsychotic treatment [19, 20]. It has been reported that berberine improved the cognitive impairments resulting from diabetes for its neuroprotection effects in animal experiments [21]. Studies on animal models also found berberine’s positive effects on depressive-like behavior [22]. It is believed that the core mechanism of this efficacy lies in its normalization of the pro-inflammatory factors [23]. To verify this hypothesis and explore for a new therapeutic targets, in this study we aimed (i) to determine the efficacy of berberine on negative symptoms and cognitive impairments in patients with chronic schizophrenia; (ii) and measure berberine anti-inflammatory effects via IL-1β, IL-6 and TNF-α markers.

## Methods

### Study design

Between January 2020 and December 2021, a randomized, 3-month, open-label, and parallel-group trial was conducted at three hospitals: Kangci Hospital of Jiaxing, Second Specialized Hospital of Hegang, and Second People’s Hospital of Tongxiang, and Second People’s Hospital of Taizhou.

Patients who met the diagnostic criteria for schizophrenia according to the International Statistical Classification of Diseases and Related Health Problems (Tenth Edition) (ICD-10) and the Mini-International Neuropsychiatric Interview (MINI) were eligible for this trial. Inclusion criteria were as follows: (1) at least 10 years duration of schizophrenia [24]; (2) a minimum required severity of negative symptoms defined as a score of ≥ 3 on at least two of the five global rating items (items 7, 12, 16, 21 or 24) of Scale for the Assessment of Negative Symptoms (SANS) [25], (selecting patients that have negative symptoms as a dominant clinical feature); (3) no obvious positive symptoms defined as a score of < 3 on each of the four global rating items (items 7, 19, 25 and 34) of Scale for the Assessment of Positive Symptoms (SAPS) [26]; (4) Hamilton Depression Scale-17 Items (HAMD-17) score ≤ 7 [27], and Hamilton Anxiety Scale (HAMA) ≤ 7 [28] (to exclude patients with symptoms of depression and anxiety); (5) participants aged 16 to 60 years with sufficient fluency in Chinese language to complete study procedures. Exclusion criteria were as follows: (1) cognitive impairments or negative symptoms due to other diseases; (2) patients receiving first-generation antipsychotics due to the possibility of drug-induced negative symptoms [29, 30]; (3) history of neurologic diseases; (4) any other serious mental illness other than schizophrenia as a primary diagnosis; (5) participants with clear inflammatory conditions due to infection, autoimmune diseases or other reasons; (6) or any other unstable medical diagnosis; (7) contraindications of berberine.

Using a computer-generated random allocation sequence, all participants were assigned to the experimental group (receiving berberine) or control group (receiving placebo tablet) in 1:1 ratio. The dosage of berberine was determined as 300 mg three times a day according to previous study conducted by Li et al. [31]. Placebo tablet was composed of amylodextrin and food coloring substance that has the same size, shape, mass, and color as berberine tablet. What needed to be pointed out was that our trial was performed on the basis of these enrolled patients’ previous therapy, especially the atypical antipsychotics.

This trial was approved by the ethics committees of the Kangci Hospital of Jiaxing, Second Specialized Hospital of Hegang, Second People’s Hospital of Tongxiang, and Second People’s Hospital of Taizhou. The clinical trial registration number is ChiCTR2000035542. All patients or their legal guardians provided written informed consent on trial participation.

### Clinical assessment of negative symptoms

The severity of negative symptoms was rated with SANS [25] which includes 25 items divided into five sub-scales; these aimed to evaluate separately apathy, poverty of thought, abulia, lack of interest and social interaction, and attention dysfunction. Each item is rated on an ordinal scale from 0 to 5, corresponding to the normal, suspicious, mild, moderate, obvious, and severe states, respectively. There are three common ways to calculate SANS scores. One is the summation of all items, resulting in a score ranging from 0–120; the second is the total score of five global rating items (item 7, 12, 16, 21 or 24) resulting in scores ranging from 0–25; the last is the sub-scores of five global rating items which determine the severity of apathy, poverty of thought, abulia, lack of interest and social interaction, and attention dysfunction, respectively. Higher scores indicate more severe negative symptoms. The subtype of schizophrenia that mainly manifested with negative symptoms is defined as a score of ≥ 3 on at least two of the five global rating items. All three assessment methods were reported in this study [25].

### Cognitive function assessment

The Trail-making Test A (TMT-A) was used to assess the attention and processing speed. This test consists of 25 consecutive numbers randomly arranged on an A4 page. Subjects are asked to draw a line that connects numbers in a sequence from 1 to 25 within 150 s. The total time (in seconds) spent to complete the task is recorded as the score. A TMT-A score ≥ 72.5 s is considered as having impairment in attention and processing speed [32].

To accommodate elderly participants with limited knowledge of English alphabet, the Chinese version of Trail-making Test B (TMT-B) was used to assess the executive function. The Chinese version of TMT-B consists of 25 numbers enclosed in 13 circles (from ) and 12 squares (from ), which are randomly arranged on an A4 page. Subjects were asked to draw a line to connect numbers in circles and squares (e.g. ) within 300 s. The time spent in accomplishing the task (in seconds) is recorded as the test score. The TMT-B score ≥ 135.5 s is considered as having impairment in executive function [32].

The time difference (TMT-B minus TMT-A) was used to assess the disturbance variable which indicated the ability of diversion from one target to another. The greater difference in scores between two tasks indicates a greater disturbance variable and worse diversion ability [32].

The Hopkins Verbal Learning Test (HVLT) score consists of total learning score and recognition score. We only chose the part of the total learning score to reveal patients’ immediate memory. The HVLT includes 12 nouns and the individuals are required to recall the nouns immediately with no limitation on sequence after the examiner reads these words out loud. The time interval of each noun read by the examiner is 2 s. This procedure is repeated three times, and the number of correct recalls is recorded as the total score (ranging from 0 to 36). The total learning score of HVLT ≤ 21.5 is considered as having impairment in immediate memory functioning [33].

### Inflammatory factors

For each participant, a total of 5 mL venous blood was obtained following 6-h fasting. Blood samples were incubated until complete coagulation; next centrifuged for 10 min at 3500 r/min. The resultant serum was used directly for subsequent assays. Serum levels of IL-1β, IL-6, and TNF-α were determined using enzyme linked immunosorbent assay (Enzyme-linked Biotechnology Co., Ltd., Shanghai, China) according to the manufacturer’s protocol.

### Primary and secondary study outcomes

The between-group difference of SANS was considered as primary outcome for the present study. Other clinical measures were used as secondary outcomes. Of note, the results of the study outcomes had no impact on the clinical decision regarding the diagnostic status of study participants. All the outcome measures were assessed or determined at baseline and each month after treatment (month 1, month 2, and month 3).

### Statistical analysis

The sample size calculation assumed a therapeutic effect of 10 and a standard deviation (SD) of 12 in SANS [34]; factoring a significance level of 5% and study power of 80%, with drop-out rate of 20%, the present analysis required at least 42 participants in each study group. Statistical analyses were conducted using SPSS v25.0 (IBM SPSS, Armonk, NY). Quantitative data were expressed as mean (SD), and categorical variables were expressed as frequency (%). The per-protocol analysis was chosen for this study. Given that the data were normally distributed (Kolmogorov–Smirnov tests), thus the demographic and clinical characteristics of patients were analyzed using the t-test and Chi-squared test; between-group differences were examined using the independent-samples t-test; repeated measures analysis of variance (ANOVA) was used to evaluate the changes in outcome measures across the treatment (month 3 minus baseline). The Pearson correlation analysis was used to analyze the correlations between the changes of inflammation markers’ concentrations and those of clinical outcome measures after treatment (month 3 minus baseline) in the experimental group. Multiple linear regression was used to analyze the influence factors, with the change of the total score of SANS, TMT-A and TMT-B as dependent variables (month 3 minus baseline), changes of IL-1β, IL-6 and TNF-α as independent variables (month 3 minus baseline), and the age, sex, education, duration of illness and duration of treatment as covariates. P values < 0.05 were considered indicative of statistical significance, and mean differences (MD) and 95% confidence interval (CI) were also listed.

## Results

### Demographic and clinical characteristics

**Fig. 1:**
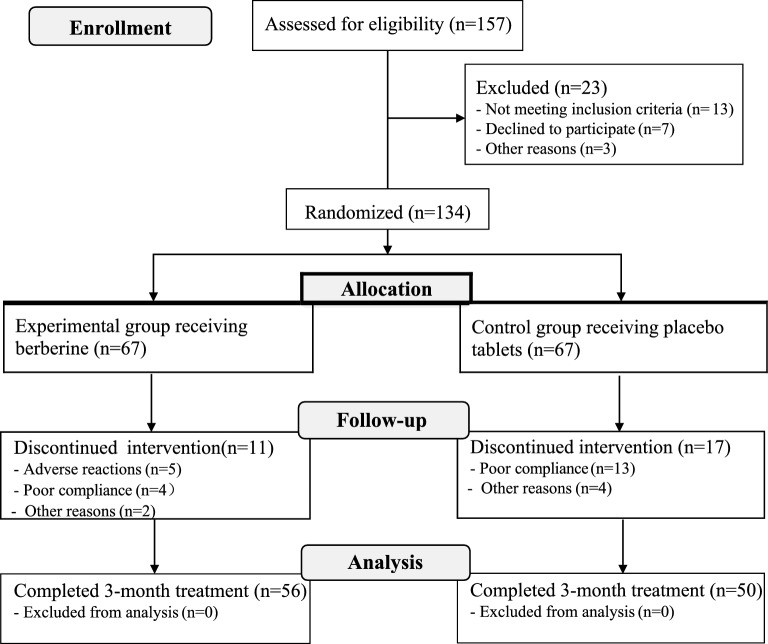
Flow diagram of the study subjects

Among the 157 participants who presented for eligibility assessment, 134 qualified for the study per inclusion criteria, allocating 67 participants for either treatment or placebo study group. During the treatment, 11 patients in the experimental group and 17 patients in the control group withdrew from the trial before its completion due to adverse reactions, poor compliance with treatment, or other reasons (loss of contact, newly emerging physical conditions, and fluctuation of psychiatric symptoms). Therefore 106 participants completed the study. Data from 56 patients who received berberine and 50 patients who received placebo were included in the final analyses. The flow diagram of patient enrollment is provided in Fig. 1. The demographic and clinical features of the patients who completed the trial were summarized in Table 1. The two groups had no statistical differences regarding the demographic or clinical characteristics at baseline (all *P* > 0.05).

**Table 1:** Demographic and clinical characteristics of the participants who completed the study

Parameter	Exp	Con	MD/RD (95% CI)	t/χ^2^	P
Age,* y*	42.71 ± 7.86	44.82 ± 8.09	− 2.11 (− 5.17 to 0.95)	− 1.352	0.179
Sex (M/F)	23/33	21/29	− 0.93% (− 19.72% to 17.86%)	0.010	0.923
Education (P/M)	21/35	24/26	− 10.50% (− 29.28% to 8.28%)	1.192	0.275
Duration of Illness, *m*	166.20 ± 33.66	173.18 ± 27.47	− 6.98 (− 18.87 to 11.91)	− 1.151	0.252
SAPS	4.29 ± 1.97	4.36 ± 2.02	− 0.07 (− 0.79 to 0.65)	− 0.190	0.850
HAMA	2.79 ± 2.17	2.90 ± 2.12	− 0.11 (− 0.91 to 0.69)	− 0.271	0.787
HAMD-17	2.88 ± 2.35	2.78 ± 2.19	0.10 (− 0.83 to 1.03)	0.212	0.832

M/F indicates male/female; P/M indicates primary school/middle school or higher levels; Exp indicates the experimental group; Con indicates the control group; MD indicates mean difference; RD indicates rate difference; CI indicates confidence interval

### Negative symptoms

The total score of SANS was significantly lower in patients who received berberine compared to placebo at the 2nd and 3rd month of treatment (MD − 8.18, 95% CI − 15.42 to − 0.94, t = − 2.215, *P* = 0.029; MD − 10.06, 95% CI − 17.30 to − 2.82, t = − 2.722, *P* = 0.008; respectively). The total score of five global rating items of SANS was significantly lower in patients who received berberine compared to placebo at the 3rd month of treatment (MD − 2.32, 95% CI − 4.07 to − 0.57, *t* = − 2.597, *P* = 0.011). As per each subscale of SANS, the score of attention dysfunction was significantly lower in patients who received berberine compared to placebo at the 2nd and 3rd of treatment (MD − 0.63, 95% CI − 1.14 to − 0.12, *t* = − 2.438, *P* = 0.016; MD − 1.03, 95% CI − 1.53 to − 0.53, *t* = − 4.006, *P* < 0.001; respectively); while there were no significant differences at the score of apathy, poverty of thought, abulia, and lack of interest and social interaction (all *P* > 0.05). From baseline to month 3, there was a significant group × time effect in the total score of SANS between the two groups (*F* = 3.887; *P* = 0.015). The details were listed in Table 2.

**Table 2:** Assessments of negative symptoms and cognitive function, and serum levels of inflammation markers

Out measures	Exp	Con	MD (95% CI)	*t*	*P*	*F(group* × *time)*	*P*
Total score of SANS							
M0	80.82 ± 19.25	84.78 ± 18.41	− 3.96 (− 11.22 to 3.30)	− 1.069	0.288	3.887	**0.015**
M1	78.79 ± 19.52	84.60 ± 18.49	− 5.81 (− 13.14 to 1.52)	− 1.554	0.123		
M2	76.88 ± 19.26	85.06 ± 18.29	− 8.18 (− 15.42 to − 0.94)	− 2.215	**0.029**		
M3	75.04 ± 18.96	85.10 ± 18.66	− 10.06 (− 17.30 to − 2.82)	− 2.722	**0.008**		
Total score of five global rating items of SANS							
M0	15.91 ± 4.37	16.66 ± 4.72	− 0.75 (− 1.64 to 0.14)	− 0.841	0.402	2.383	0.081
M1	15.41 ± 4.41	16.54 ± 4.80	− 1.13 (− 2.90 to 0.64)	− 1.251	0.214		
M2	14.80 ± 4.34	16.54 ± 4.78	− 1.74 (− 3.50 to 0.02)	− 1.940	0.055		
M3	14.20 ± 4.32	16.52 ± 4.81	− 2.32 (− 4.07 to − 0.57)	− 2.597	**0.011**		
Apathy of SANS							
M0	2.95 ± 1.47	3.18 ± 1.29	− 0.23 (− 0.76 to 0.30)	− 0.857	0.394	1.420	0.249
M1	2.88 ± 1.48	3.10 ± 1.35	− 0.22 (− 0.75 to 0.31)	− 0.809	0.421		
M2	2.75 ± 1.44	3.08 ± 1.34	− 0.33 (− 0.87 to 0.21)	− 1.205	0.231		
M3	2.66 ± 1.44	3.06 ± 1.36	− 0.40 (− 0.94 to 0.14)	− 1.446	0.151		
Poverty of thought of SANS							
M0	3.27 ± 1.34	3.40 ± 1.34	− 0.13 (− 0.64 to 0.38)	− 0.501	0.617	1.856	0.150
M1	3.21 ± 1.31	3.38 ± 1.32	− 0.17 (− 0.73 to 0.39)	− 0.596	0.552		
M2	3.13 ± 1.27	3.42 ± 1.31	− 0.29 (− 0.78 to 0.20)	− 1.164	0.247		
M3	3.07 ± 1.28	3.44 ± 1.30	− 0.37 (− 0.87 to 0.13)	− 1.456	0.148		
Abulia of SANS							
M0	3.39 ± 1.01	3.48 ± 1.12	− 0.09 (− 0.51 to 0.33)	− 0.417	0.677	1.371	0.263
M1	3.29 ± 1.08	3.50 ± 1.10	− 0.21 (− 0.62 to 0.20)	− 1.001	0.319		
M2	3.23± 1.12	3.50 ± 1.08	− 0.27 (− 0.70 to 0.16)	− 1.239	0.218		
M3	3.18± 1.14	3.46 ± 1.10	− 0.28 (− 0.71 to 0.15)	− 1.281	0203		
Lack of interest and social interaction of SANS							
M0	3.38 ± 1.03	3.50 ± 1.10	− 0.12 (− 0.51 to 0.27)	− 0.599	0.550	1.449	0.241
M1	3.27 ± 1.14	3.46 ± 1.08	− 0.19 (− 0.61 to 0.23)	− 0.879	0.382		
M2	3.25 ± 1.15	3.46 ± 1.02	− 0.21 (− 0.63 to 0.21)	− 0.977	0.331		
M3	3.21 ± 1.15	3.44 ± 1.00	− 0.23 (− 0.65 to 0.19)	− 1.063	0.290		
Attention dysfunction of SANS							
M0	2.93 ± 1.40	3.10 ± 1.27	− 0.17 (− 0.68 to 0.34)	− 0.651	0.516	2.661	0.059
M1	2.80 ± 1.32	3.10 ± 1.30	− 0.30 (− 0.81 to 0.21)	− 1.154	0.251		
M2	2.45 ± 1.32	3.08 ± 1.32	− 0.63 (− 1.14 to − 0.12)	− 2.438	**0.016**		
M3	2.09 ± 1.33	3.12 ± 1.29	− 1.03 (− 1.53 to − 0.53)	− 4.006	** < 0.001**		
TMT-A, *s*							
M0	68.82 ± 25.35	71.74 ± 22.95	− 2.92 (− 12.27 to 6.43)	− 0.612	0.542	3.317	**0.028**
M1	67.44 ± 26.08	71.81 ± 22.94	− 4.37 (− 13.87 to 5.13)	− 0.902	0.369		
M2	62.73 ± 24.47	72.39 ± 22.96	− 9.66 (− 18.81 to − 0.51)	− 2.070	**0.041**		
M3	60.67 ± 22.84	71.31 ± 23.53	− 10.64 (− 19.56 to − 1.72)	− 2.338	**0.021**		
TMT-B, *s*							
M0	138.48 ± 51.01	138.42 ± 50.02	0.06 (− 19.54 to 19.66)	0.006	0.995	3.054	**0.037**
M1	134.32 ± 50.90	137.53 ± 49.94	− 3.21 (− 22.63 to 16.21)	− 0.324	0.746		
M2	126.06 ± 48.89	136.00 ± 48.64	− 9.94 (− 28.92 to 9.04)	− 1.037	0.302		
M3	122.14 ± 47.53	135.09 ± 49.37	− 12.95 (− 31.57 to 5.67)	− 1.363	0.176		
Time difference (TMT-B minus TMT-A), *s*							
M0	69.66 ± 30.63	66.68 ± 29.11	2.98 (− 8.43 to 14.39)	0.512	0.610	0.934	0.432
M1	66.88 ± 30.10	65.72 ± 29.23	1.16 (− 10.15 to 12.47)	0.201	0.841		
M2	63.34 ± 30.32	63.61 ± 28.50	− 0.27 (− 11.53 to 10.99)	− 0.047	0.963		
M3	61.47 ± 30.71	63.78 ± 28.70	− 2.31 (− 13.66 to 9.04)	− 0.399	0.691		
HVLT							
M0	20.32 ± 7.16	18.62 ± 7.54	1.70 (− 1.13 to 4.53)	1.178	0.241	0.361	0.781
M1	20.50 ± 7.17	18.58 ± 7.56	1.92 (− 0.91 to 4.75)	1.328	0.187		
M2	20.73 ± 7.09	18.72 ± 7.75	2.01 (− 0.84 to 4.86)	1.383	0.170		
M3	20.95 ± 7.33	18.92 ± 8.01	2.03 (− 0.92 to 4.98)	1.347	0.181		
IL-1β, *ng/l*							
M0	67.01 ± 19.94	70.46 ± 20.61	− 3.45 (− 11.25 to 4.35)	− 0.867	0.388	6.556	** < 0.001**
M1	61.50 ± 19.07	69.75 ± 19.97	− 8.25 (− 15.75 to − 0.75)	− 2.155	**0.033**		
M2	59.24 ± 18.56	68.65 ± 19.19	− 9.41 (− 16.67 to − 2.15)	− 2.541	**0.013**		
M3	55.52 ± 17.11	69.23 ± 19.65	− 13.71 (− 20.78 to − 6.64)	− 3.802	** < 0.001**		
IL-6, *ng/l*							
M0	39.86 ± 14.86	40.98 ± 13.91	− 1.12 (− 6.64 to 4.40	− 0.398	0.692	8.326	** < 0.001**
M1	35.38 ± 11.67	40.77 ± 12.57	− 5.39 (− 10.05 to -0.73)	− 2.265	**0.026**		
M2	34.72 ± 11.58	41.00 ± 12.20	− 6.28 (− 10.85 to − 1.71)	− 2.693	**0.008**		
M3	32.52 ± 10.41	40.62 ± 11.55	− 8.10 (− 13.32 to − 3.88)	− 3.762	** < 0.001**		
TNF-α, *ng/l*							
M0	48.90 ± 17.13	46.57 ± 14.90	2.33 (− 3.90 to 8.56)	0.733	0.465	5.282	**0.003**
M1	44.49 ± 15.38	47.67 ± 14.22	− 3.18 (− 8.89 to 2.53)	− 1.091	0.278		
M2	41.89 ± 15.38	48.01 ± 13.65	− 6.12 (− 11.60 to − 0.64)	− 2.189	**0.031**		
M3	37.70 ± 13.95	48.59 ± 14.03	− 10.89 (− 16.28 to − 5.50)	− 3.963	** < 0.001**		

*M*_0_ indicates baseline; *M*_1_ Month 1 of treatment; *M*_2_ Month 2 of treatment; *M*_3_ Month 3 of treatment

### Cognitive function

Concerning the attention and processing speed, the score of TMT-A was significantly lower in patients who received berberine compared to placebo at the 2nd and 3rd month of treatment (MD − 9.66, 95% CI − 18.81 to − 0.51, *t* = − 2.070, *P* = 0.041; MD − 10.64, 95% CI − 19.56 to − 1.72, *t* = − 2.338, *P* = 0.021; respectively). From baseline to month 3, there was a significant group × time effect in the score of TMT-A between the two groups (F = 3.317; *P* = 0.028). There were no significant differences between two groups in executive functioning (the score of TMT-B) at each time point (all *P* > 0.05), but there was a significant group × time effect in the score of TMT-B between the two groups (F = 3.054; *P* = 0.037) from baseline to month 3. Concerning the ability of diversion from one target to another, there were no significant differences at each time point in the time difference (TMT-B minus TMT-A) (all *P* > 0.05). As to immediate memory, there were also no significant differences at each time point in the score of HVLT (all *P* > 0.05). The details were listed in Table 2.

### Inflammatory factors

At 1, 2 and 3 months of treatment, the serum level of IL-1β was significantly lower in patients who received berberine compared to placebo (MD − 8.25, 95% CI − 15.75 to − 0.75, *t* = − 2.155, *P* = 0.033; MD − 9.41, 95% CI − 16.67 to − 2.15, *t* = − 2.541, *P* = 0.013; MD − 13.71, 95% CI − 20.78 to − 6.64, *t* = − 3.802, *P* < 0.001; respectively). Similar effects were identified for IL-6 marker as berberine treatment group showed significantly lower serum levels as compared to placebo group (MD -5.39, 95% CI − 10.05 to − 0.73, *t* = − 2.265, *P* = 0.026; MD − 6.28, 95% CI − 10.85 to − 1.71, *t* = − 2.693, P = 0.008; MD − 8.10, 95% CI − 13.32 to − 3.88, *t* = − 3.762, *P* < 0.001; respectively). The serum level of TNF-α was significantly lower in patients who received berberine compared to placebo at the 2nd and 3rd month of treatment (MD − 6.12, 95% CI − 11.60 to − 0.64,* t* = − 2.189, *P* = 0.031; MD − 10.89, 95% CI − 16.28 to − 5.50, *t* = − 3.963, *P* < 0.001; respectively). From baseline to month 3, there were significant group × time effects in the serum level of IL-1β, IL-6 and TNF-α between the two groups (*F* = 6.556, *P* < 0.001; *F* = 8.326, *P* < 0.001; *F* = 5.282, *P* < 0.003; respectively). The details were listed in Table 2.

### Associations between inflammatory factors and negative symptoms and cognitive function

The correlations between the changes of serum inflammatory factors’ concentrations and those of clinical measures (month 3 minus baseline) in the experimental were displayed in Table 3. With respect to negative symptoms, there was a significant positive correlation between the change of serum IL-1β level and the change of total score of SANS (r = 0.210, *P* = 0.039). With respect to the attention and processing speed, there were significant positive correlations between the change of serum IL-1β level and the change of score of TMT-A (*r* = 0.522, *P* < 0.001); between the change of serum IL-6 level and the change of score of TMT-A (*r* = 0.562, *P* < 0.001). With respect to executive function, there was a significant positive correlation between the change of serum IL-1β level and the change of score of TMT-B (r = 0.811, P < 0.001); between the change of serum IL-6 level and the change of score of TMT-B (r = 0.664,* P* < 0.001); between the change of serum TNF-α level and the change of score of TMT-B (r = 0.472, *P* < 0.001). With respect to the ability of diversion from one target to another, there was a significant positive correlation between the change of serum IL-1β level and the change of time difference (TMT-B minus TMT-A) (r = 0.289, *P* = 0.031). Regarding the immediate memory evaluation, there were no significant correlations between the changes of serum levels of inflammatory factors and the change of score of HVLT (all *P* > 0.05). The Pearson correlation analysis showed that there were no significant correlations between the changes of serum levels of inflammatory factors and those of clinical outcome measures (month 3 minus baseline) in the control group (all *P* > 0.05).

**Table 3:** Correlations between the changes of inflammatory factors’ concentrations and those of clinical measures after treatment (month 3 minus baseline) in the experimental group

Out measures	IL-1β			IL-6			TNF-α		
	r	t	P	r	t	P	r	t	P
Total score of SANS	0.210	2.112	**0.039**	0.078	1.578	0.120	0.023	1.168	0.248
Total score of five global rating items of SANS	0.041	0.303	0.763	0.033	0.245	0.807	0.004	0.026	0.979
Apathy of SANS	− 0.001	− 0.008	0.994	0.014	0.100	0.921	0.007	0.052	0.959
Poverty of thought of SANS	0.007	0.050	0.960	-0.002	-0.011	0.991	0.001	0.004	0.997
Abulia of SANS	− 0.008	− 0.056	0.956	− 0.007	− 0.045	0.964	− 0.009	− 0.067	0.947
Lack of interest and social interaction of SANS	− 0.001	− 0.005	0.996	− 0.005	− 0.040	0.968	− 0.002	− 0.013	0.990
Attention dysfunction of SANS	0.045	0.332	0.741	0.035	0.257	0.798	0.014	0.105	0.917
TMT-A	0.522	4.497	** < 0.001**	0.562	4.994	** < 0.001**	0.227	1.714	0.092
TMT-B	0.811	10.196	** < 0.001**	0.664	6.521	** < 0.001**	0.472	3.930	** < 0.001**
TMT-B minus TMT-A	0.289	2.220	**0.031**	0.102	0.751	0.456	0.244	1.852	0.069
HVLT	− 0.072	− 0.528	0.600	− 0.045	− 0.330	0.743	− 0.023	− 0.169	0.866

*r* indicates correlation coefficient

Patients in the berberine group were analyzed by multiple linear regression (Table 4). With respect to the negative symptoms assessed by the total score of SANS (model *R*^*2*^ = 0.267, *F* = 4.378,* P* = 0.017), the change of serum IL-1β level (month 3 minus baseline) were the independent influencing factors (*b* = 0.112, *P* = 0.036). With respect to the attention and processing speed assessed by TMT-A (model *R*^*2*^ = 0.442,* F* = 5.531, *P* = 0.007), the changes of serum IL-1β and IL-6 levels (month 3 minus baseline) were the independent influencing factors (*b* = 0.567, *P* < 0.001; *b* = 0.593, *P* < 0.001; respectively). With respect to the executive function assessed by TMT-B (model *R*^*2*^ = 0.681,* F* = 5.9608, *P* = 0.005), the changes of serum IL-1β, IL-6 and TNF-α (month 3 minus baseline) were the independent influencing factors (*b* = 0.983, *P* < 0.001;* b* = 0.720, *P* < 0.001; *b* = 0.317, *P* = 0.014; respectively).

**Table 4:** Analysis of factors related to the changes (month 3 minus baseline) of negative symptoms and cognitive function in the experimental group

Variables	Negative symptoms (R^2^ = 0.267)			Attention and processing speed (R^2^ = 0.442)			Executive function (R^2^ = 0.681)		
	b	t	P	b	t	P	b	t	P
IL-1β, *ng/l*	0.112	2.157	**0.036**	0.567	4.562	** < 0.001**	0.983	11.815	** < 0.001**
IL-6, *ng/l*	0.034	1.452	0.153	0.593	5.282	** < 0.001**	0.720	6.646	** < 0.001**
TNF-α, *ng/l*	0.027	1.286	0.205	0.177	1.628	0.110	0.317	2.561	**0.014**

*b* indicates partial regression coefficient; R^2^ indicates adjusted coefficient of determination; female was defined as the control in the variable of sex; negative symptoms was assessed by the total score of SANS; attention and processing speed was assessed by TMT-A; executive function was assessed by TMT-B

### Adverse reactions to berberine

Safety analyses included all enrolled patients (n = 134). Adverse reactions induced by previous therapy such as antipsychotics were not reported. The most frequent adverse reactions to berberine were nausea (5 cases), mild stomach pain (7 cases), and constipation (2 case), which were resolved with time and tolerated well by the participants. However, a total of 5 patients that started berberine treatment trial had to discontinue due to adverse reactions.

## Discussion

Results from our 3-month trial showed that compared to placebo, berberine treatment improved negative symptoms, as indicated by a reduction in SANS scores (including the total score of SANS and sum of five global rating items of SANS). However, regarding the subclinical clusters of negative symptoms, the attention dysfunction became the only one to be improved after berberine treatment rather than apathy, poverty of thought, abulia, and lack of interest and social interaction. With respect to cognitive function, attention and processing speed and executive function were the two cognitive metrics that showed improvements following berberine treatment. It was worth mentioning that Kern et al.’s study considered processing speed and working memory (one component of executive function) as the most severely impaired cognitive domains in patients with schizophrenia [35]. Both negative symptoms and cognitive functioning improvements coincided with significant decrease in serum inflammatory markers, IL-1β, IL-6 and TNF-α.

There were more positive outcomes according to correlation analysis such as the positive correlations between changes of inflammatory markers (IL-1β, IL-6 and TNF-α) and changes of negative symptoms and those of attention and processing speed and executive function after berberine administration. The results of multiple linear regression analysis showed that the changes of inflammatory markers (IL-1β, IL-6 and TNF-α) were the influence factors of negative symptoms and cognitive domains of attention and processing speed and executive function.

There has been an elevation in research interest in studying neuroinflammation in schizophrenia. The inflammatory cytokines in peripheral blood can penetrate the blood brain barrier to continue the inflammatory cascade in the central nervous system (CNS) [36]. It is reported that the inflammatory cytokines have closer correlations with the negative symptoms and cognitive impairments rather than positive symptoms [37, 38]. Preclinical studies using animal models consistently demonstrated that overexpression of IL-1β and IL-6 can inhibit hippocampus-dependent learning and memory functioning [39, 40]. As to the negative symptoms, previous reports showed a positive correlation between IL-6 levels and the severity of attention deficit and psychomotor retardation [41, 42]. Furthermore, the injection of IL-1β and TNF-α can lead to social withdrawal and anhedonia [43]. And some scholars even considered TNF-α as a specific inflammatory marker related to the negative symptoms [44].

Several clinical studies have proved berberine’s anti-inflammatory effects [45]. It down-regulates the expression of pro-inflammatory factors through inhibiting nuclear factor-kappaB and signaling pathways of mitogen-activated protein kinase [46, 47]. However, its clinical utility for treating neurological and psychiatric illnesses has been only recently identified. There have been several animal experiments to explore the improvement effects of berberine on cognitive impairments resulting from diabetes [21], Alzheimer’s disease [48], Huntington’s disease [49], cerebral vascular disorder [50], and schizophrenia [51]. A handful of clinical trials with schizophrenic patients showed that berberine could improve information processing speed, working memory, and social cognition [52]. For example, another animal study demonstrated the beneficial effects of berberine on depression-like behavior in a rat diabetes model [22]. The results of the present study are in consonance with a recent clinical report by Li et al. Their study also suggested that berberine may improve negative symptoms in patients with schizophrenia via anti-inflammatory action as the negative symptoms improvements coincided with significant decrease in serum inflammatory markers including IL-6 and C-creative protein [31]. However, Lie et al. reported negative subscale of the Positive and Negative Syndrome Scale (PANSS) to evaluate negative symptoms, which had fewer items (7 items) than SANS (24 items) and didn’t list the concrete types of negative symptoms compared to SANS. To add value to the previous report we also explored the efficacy of berberine on the cognitive impairments in schizophrenia.

Both clinical and animal studies converge on the notion that salutary effects of berberine are exerted due to anti-inflammatory action. Besides the mechanism of anti-inflammation, recent studies found that berberine could get across the brain-blood barrier without any chemical modification [23] and accumulate in the hippocampus to improve cognitive function via lowering the activity of prolyl oligopeptidase and cholinesterase [53]. Accumulated berberine in the hippocampus could further up-regulate the expression of brain-derived neurotrophic factor [22] and ameliorate oxidative stress [54]. The exact mechanism of action of berberine in treating psychiatric illnesses remains to be fully understood. It can be hypothesized that berberine accumulation in CNS can inhibit the activity of monoamine oxidase A [55] in adrenergic neurons and monoamine oxidase B [56] in serotonergic neurons to enhance levels of DA. These neurochemical changes may likely account for clinical improvements in negative symptoms in patients with schizophrenia.

The results of the present study should be interpreted in light of several key limitations. Our clinical trial was conducted on a small sample size with a relatively short follow-up period. We examined a narrow range of peripheral inflammatory cytokines, but data from direct cerebral inflammatory markers could have added interpretative value in advanding our understanding of the therapeutic action of berberine. Another important limitation to consider is the fact that the study participants did not receive a uniform antipsychotic treatment before entering the clinical trial with berberine as an adjunctive treatment agent. However, the outcomes of our trial support the application of berberine as an adjuvant drug to improve the negative symptoms and cognitive impairments in patients with chronic schizophrenia. Future studies should replicate these findings on larger sample size.

## Conclusions

Results of the current study showed that berberine mitigated the negative symptoms and cognitive deficits in patients with chronic schizophrenia. This treatment regimen is safe and may work through anti-inflammatory effect. Therefore, it may be a valuable complementary and an alternative therapeutic option for chronic schizophrenia with residual negative symptoms and cognitive deficits.

## Data Availability

The datasets generated and analyzed in the present study are available from the corresponding author upon reasonable request.

## Abbreviations

SANS: Assessment of negative symptoms
TMT-A: Trail-making Test A
TMT-B: Trail-making Test B
HVLT: Hopkins Verbal Learning Test
IL-1β: Interleukin-1β
IL-6: Interleukin-6
TNF-α: Tumor necrosis factor-α
MD: Mean differences
CI: Confidence interval
CNS: Central nervous system
DA: Dopamine
